# Particulate Matter concentration measurement in ambient air: analysis of filter material and recovery time variables for uncertainty assessment

**DOI:** 10.1007/s10661-026-15542-4

**Published:** 2026-06-11

**Authors:** Lorenzo Raso, Maria Rosaria Della Rocca, Vincenzo Belgiorno, Vincenzo Naddeo, Tiziano Zarra

**Affiliations:** 1 Sanitary Environmental Engineering Division (SEED), Department of Civil Engineering, University of Salerno, Via Giovanni Paolo II 132, 84084 Fisciano, SA Italy; 2 Regione Campania UOD, 50 06 04, Via Roberto Bracco, 15/A – 80113, Naples, NA Italy

**Keywords:** EU Air Quality Directive (2024/2881), Gravimetric analysis, Environmental monitoring, QA procedures, Air pollution assessment

## Abstract

Accurate determination of PM concentrations is essential to ensure compliance with regulatory thresholds and to support effective strategies for air-quality management and health protection. In Europe, gravimetric analysis according to EN12341:2023 is the reference method for quantifying PM10 and PM2.5 concentrations in ambient air. While the standard acknowledges the presence of measurement uncertainty, it does not explicitly address the specific contributing variables. This study presents and discusses the systematic evaluation of the contribution of non-standard variables to measurement uncertainty in PM10 and PM2.5 determinations. Three major categories of interference (*environmental, operational, and instrumental*) were identified, together with related uncertainty indicators. Experimental campaigns were conducted to assess the influence of different filter materials and filter recovery times under real in-situ conditions. Results revealed that filter recovery time contributes to an uncertainty of 18.09% in PM2.5 concentration measurements, whereas filter material accounts for an uncertainty of 4.85% in PM10 determinations. Overall, the analyses reveal a consistently higher sensitivity of the finer particulate fraction to the uncertainty factors investigated. Within the broader framework of environmental monitoring and assessment, this study strengthens the scientific basis for the design and evaluation of air-quality monitoring systems supporting exposure assessment and pollution risk analysis. These findings contribute to improving data quality, comparability and robustness in monitoring networks, with direct implications for the design and development of advanced monitoring systems, sampling strategies, network optimization and quality assurance procedures.

**Graphical Abstract:**

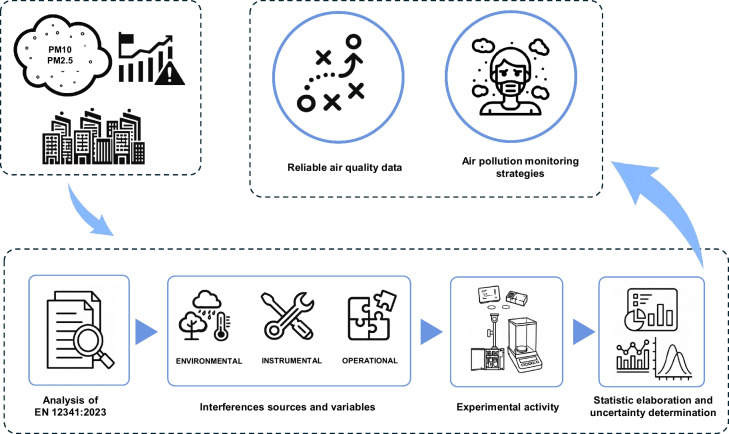

## Introduction

Air pollution is a critical environmental challenge for modern society due to its pervasive adverse effects on human health, ecosystems, and atmospheric processes (Beloconi & Vounatsou, 2023).

Atmospheric pollutants are commonly divided into conventional, such as particulate matter (PM), nitrogen oxides (NOX), sulfur dioxide (SO), carbon monoxide (CO), and ozone (O3), and non-conventional pollutants, including odorous or semi-volatile compounds, which are increasingly being recognized as significant for environmental quality, especially in urban and industrial settings (Fowler et al., 2020; Higham et al., 2025; Ibarra-Espinosa et al., 2022; Oliva et al., 2024; Zarra et al., 2022). The increasing burden of disease associated with particulate exposure has led the World Health Organization to revise its Global Air Quality Guidelines (Hoffmann et al., 2021). In Europe, the recently adopted Directive (EU) 2024/2881 further tightens the limit values for atmospheric pollutants (European Commisson, 2024). Under these stricter regulatory thresholds, even small deviations caused by measurement uncertainty may critically influence compliance assessments (European Environment Agency, 2022; Lipfert, 1994).

Among conventional pollutants, PM serves as a primary indicator of air quality, given its strong association with mortality and morbidity (Naddeo et al., 2012). Exposure to PM2.5 above WHO guideline levels has been linked to hundreds of thousands of premature deaths in Europe (European Environment Agency, 2022). Classification of PM by aerodynamic diameter allows for characterization of both source contributions and health impacts (Agarwal et al., 2024). Coarser particles (PM10), generated by mechanical processes (construction, road dust, natural sources), are largely deposited in the upper respiratory tract and can provoke irritation and inflammatory responses (Ji et al., 2010; Thakur & Patel, 2024). Finer particles (PM2.5), originating largely from combustion (traffic, industry, heating), can penetrate to alveoli and translocate into the bloodstream, triggering systemic effects such as oxidative stress and cardiovascular disease (Attiya & Jones, 2022; Cohen et al., 2017; Kobayashi et al., 2023; Mecca et al., 2024; Ruiz-Páez et al., 2023; Xu et al., 2022). Beyond total mass concentration, PM2.5 contains a higher proportion of semi-volatile components such as ammonium nitrate and low-volatility organic compounds, prone to volatilization or adsorption depending on temperature and humidity (Chow et al., 2010; Schaap et al., 2004; Viana et al., 2006). These properties make PM2.5 inherently more vulnerable to sampling artifacts than PM10 (Patel & Aggarwal, 2022; Vecchi et al., 2009). Health outcomes therefore depend not only on ambient PM mass concentration but also on particle size distribution, chemical composition, and emission source (Fraiese et al., 2019; Young et al., 2023).

Because of its health relevance, the characterization and measurement of these PM fractions are therefore essential for effective air-quality management and public health protection (Nim et al., 2025, Ruiz-Páez et al., 2023). In this sense regulatory frameworks have progressively tightened air-quality standards. In the European Union, the recent Directive (EU) 2024/2881 revised downward the limit values for ambient concentrations of PM2.5 and PM10, aligning them more closely with the WHO Global Air Quality Guidelines and advancing the Zero Pollution goal for 2050 (Yang et al., 2022). The annual mean limit for PM2.5 has been reduced from 25 to 10 µg/m^3^ by 2030, and the PM10 limit from 40 to 20 µg/m^3^ (European), reflecting the increasing urgency of reliable and accurate air-quality assessment (Dubey et al., 2022; Hoffmann et al., 2021). Ensuring compliance with these stringent thresholds requires robust monitoring techniques capable of producing data of high quality and low uncertainty (Murray et al., 2019).

PM concentration is measured through two main approaches: offline (manual) and real-time (continuous) approaches (Korevaar et al., 2025; Ma et al., 2025; Noble et al., 2001). The offline method is based on gravimetric analysis which requires particle collection on pre-conditioned filters and subsequent laboratory weighing to calculate mass concentration over a fixed sampling interval of 24 h (Buwaniwal et al., 2024; Patel & Aggarwal, 2022). In the offline methods, sampling and determination occur in two separate phases, as the collected particulate matter is analyzed in a laboratory after the sampling period (Chung et al., 2001; Fang et al., 2024; Tasić et al., 2012). While in continuous methods, sampling and measurement take place simultaneously. These methods, such as optical or microbalance ones, allow near real-time monitoring of PM concentration provided they meet equivalence criteria against the reference method (Molaie & Lino, 2021; Shukla & Aggarwal, 2022; Zheng et al., 2025). According to EN12341:^2023^, gravimetric analysis remains the European reference method for PM10 and PM2.5 determination (Korevaar et al., 2025). Being the legally recognized Federal Reference Method, gravimetry provides the benchmark against which continuous and equivalent instruments must be evaluated (Brown et al., 2006). Nevertheless, previous intercomparison studies have revealed discrepancies between gravimetric and continuous methods due to sampling artifacts, filter handling and environmental variability (Chartier & Weitz, 1998).

EN12341:^2023^ permits the use of PTFE, quartz and glass fiber filters provided that they fulfill the required performance criteria. In contrast, the US-EPA FRM/FEM method for PM2.5 (40 CFR Part 50, Appendix L) mandates the use of PTFE substrates because of their low chemical reactivity and reduced susceptibility to volatilization and adsorption artifacts (United States Environmental Protection Agency, 1997; Kleinman et al., 2017).

This difference reflects a fundamental divergence between regulatory approaches. The European standard adopts a performance-based framework, allowing different filter media provided that measurement quality objectives and equivalence criteria are met (EN12341:^2023^; EMEP/EEA, 2019). Conversely, the U.S. framework prescribes PTFE filters to minimize known sampling artifacts, particularly those associated with semi-volatile species and gas adsorption (Chow et al., 2010; Schaap et al., 2004; Viana et al., 2006).

Under EN12341:2023 (Clause 9.3.2.11) sampling artifacts associated with adsorption/desorption processes are acknowledged as part of the method limitations and are managed through compliance with overall measurement uncertainty and equivalence performance criteria rather than being explicitly corrected within the gravimetric uncertainty budget.

In European monitoring networks, quartz and glass fiber filters are still widely used due to their compatibility with subsequent chemical analyses (e.g., carbonaceous fraction determination), which are often integrated into air-quality assessment programs (Putaud et al., 2010; EMEP/EEA, 2019). This operational flexibility, however, may introduce artifacts that are less controlled compared to PTFE-based sampling, highlighting the importance of explicitly quantifying their contribution to measurement uncertainty, as addressed in the present study.

The gravimetric method is intrinsically complex, involving a multi-step measurement chain with numerous sources of interference that may compromise accuracy. The standard EN12341:2023 acknowledges the existence of measurement uncertainty (eg. Section 9) but does not explicitly quantify the contributions of several operational aspects here referred to as “non-standard variables”, which may become relevant under real-word monitoring conditions and regulatory limits (Buonanno et al., 2011). Measurement uncertainty encompasses both random and systematic components. Random contributions include balance repeatability, temporary flow fluctuations and stochastic particle losses or gains, whereas systematic components may arise from flow-rate bias, sampler cut-off performance, incomplete conditioning, filter artefacts and electrostatic effects (Lacey & Faulkner, 2015; Zhao et al., 2013).

Inadequate control of filter storage or conditioning may exacerbate systematic deviations (Chartier & Weitz, 1998; Shukla & Aggarwal, 2022; Watson et al., 2017).

Volatilization of semi-volatile inorganic species such as ammonium nitrate (Schaap et al., 2004; Viana et al., 2006) and losses of nitrate, chloride and ammonium from quartz filters during storage (Witz et al., 1990) are known to cause negative artifacts. Conversely, adsorption of gaseous organic compounds on quartz substrates (Chow et al., 2010; Vecchi et al., 2009) and water sorption by quartz and glass filters under variable humidity (Brown et al., 2006; Widziewicz-Rzońca & Tytła, 2020) can introduce positive artifacts. Lipfert (1994) highlighted that glass filters may also promote artifacts via chemical interactions, whereas Chartier and Weitz (1998) demonstrated increased variability in PM2.5 mass when quartz rather than PTFE filters were used. Recent studies have further quantified nitrate losses in regulatory networks even when protocols are strictly followed (Chiu & Carlton, 2023).

Despite these findings, the contribution of these artifacts to total gravimetric uncertainty under real-world monitoring conditions remains insufficiently quantified.

Intercomparison exercises by the European Commission Joint Research Centre have shown larger operational variability for PM2.5 than for PM10 (Hitzenberger et al., 2004). However, no previous work has systematically isolated the influence of filter material and filter recovery time using two identical, simultaneously operating samplers under real ambient conditions (Aggarwal et al., 2013; Brown et al., 2006). Likewise, research on optical and low-cost sensors has prioritized calibration and performance metrics over detailed uncertainty analysis of operational variables (Chen et al., 2025; Dubey et al., 2022; Feng et al., 2025; Kim et al., 2011). Previous studies focus on individual artifacts (Wu et al., 2018), with limited attention to the combined effect of operational variables such as filter material and recovery time within a unified uncertainty framework, particularly in European monitoring practices.

The present study addressed this gap by developing and applying a structured experimental methodology that enables the systematic isolation and quantification of environmental, operational and instrumental contributions to uncertainty in gravimetric PM10 and PM2.5 measurements under real-world conditions. Extensive experimental activity was conducted using two identical samplers operating simultaneously to evaluate the influence of filter material and recovery time, factors not explicitly addressed in current standards but still common in practical monitoring configurations. The study focuses on quartz and glass fiber filters which are mainly used in European networks, to assess their operational uncertainty under conditions relevant to EN12341:2023.

The research advances the understanding of uncertainty in gravimetric PM measurements and provides a scientific basis for improving data reliability in regulatory monitoring networks. The findings support regulators, monitoring agencies and researchers in optimizing air-quality assessment and contribute to improving data quality, comparability and robustness in monitoring networks and quality assurance procedures.

## Material and methods

### Experimental investigation methodology

**Fig. 1:**
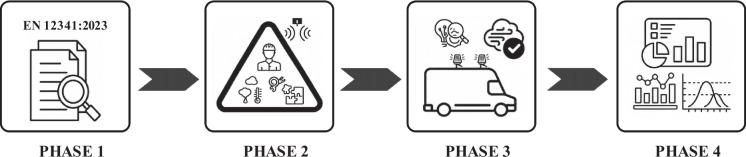
Experimental investigation methodology

The experimental investigation was structured into four sequential phases, as illustrated in Fig. 1.

The first phase consisted of a critical review of EN12341:2023. This step was essential to establish a robust methodological foundation by analyzing the normative framework in detail and identifying critical stages of the measurement chain where potential interferences may occur and compromise data reliability. The second phase focused on the identification and classification of potential interferences that could influence measurement accuracy. These interferences were systematically grouped into three main categories—environmental, operational, and instrumental—each linked to specific variables with the potential to affect gravimetric determinations. The categories and associated variables are summarized in Table 1, which highlights environmental parameters, operational conditions (including filter material and filter recovery time), and instrumental factors.

**Table 1:** Classification of identified interference categories and associated variables

Interference category	Non standard variables
Enviromental	Temperature [^o^C] Relative humidity [%]
Operational	Filter material Filter recovery time
Instrumental	Balance performance Automatic sequential sampler

In the third phase, a comprehensive experimental campaign was designed and conducted to empirically evaluate the influence of several of these variables. Repeated and simultaneous measurements were performed under controlled and representative ambient conditions, thereby allowing the isolation, comparison, and quantification of the effects of the selected variables on the gravimetric measurement process.

The fourth and final phase consisted of the estimation of uncertainties associated with the investigated non-standard variables, applying statistical approaches consistent with the principles of the *Guide to the Expression of Uncertainty in Measurement* (GUM) (Cox & O’Hagan, 2022). This phase aimed to provide quantitative insights into the contribution of each variable to the overall measurement uncertainty, thereby supporting the refinement of monitoring protocols and enhancing the robustness of gravimetric PM10 and PM2.5 assessments in compliance with reference standards.

### Experimental plan and program

Experimental activities were conducted using two identical, certified low-volume samplers (model Charlie, TCR Tecora srl, Italy), hereafter referred to as *Charlie I* and *Charlie II*. Both instruments were installed within a mobile laboratory (seedAIR, Sanitary Environmental Engineering Division, Department of Civil Engineering, University of Salerno, Italy), positioned in a dedicated parking area to ensure stable operating conditions. Both samplers, Charlie I and Charlie II, were operated at the same location during the same time period. Sampling activities were performed using two types of filter media: glass fiber filters (Ahlstrom Munksjö, Finland) and quartz fiber filters (LabExact, USA), both with a diameter of 47 mm and a nominal porosity of 2.20 µm.

The experimental campaign spanned a total duration of 168 days, structured to systematically evaluate the influence of operational interferences on the gravimetric determination of PM10 and PM2.5 concentrations. The detailed experimental plan and program and configurations adopted during the monitoring campaign, including PM fraction, filter type, recovery time, and sampler allocation, are presented in Table 2.

**Table 2:** Experimental configurations tested during the monitoring campaigns

PM fraction	Stage	Duration [days]	Sampler ID	Operative non-standard variables investigated		
				Filter material		Recovery time
PM10	I	28	Charlie I	Quartz	-	
			Charlie II	Glass		
	II	28	Charlie I	Glass	Daily	
			Charlie II		Weekly	
	III	28	Charlie I	Quartz	Daily	
			Charlie II		Weekly	
PM2.5	I	28	Charlie I	Quartz	-	
			Charlie II	Glass		
	II	28	Charlie I	Glass	Daily	
			Charlie II		Weekly	
	III	28	Charlie I	Quartz	Daily	
			Charlie II		Weekly	

The first variable investigated was the type of filter material. For both PM10 and PM2.5, parallel sampling was carried out under identical environmental conditions using glass and quartz fiber filters, enabling direct comparison of their influence on measured concentrations. The second variable examined was related to the operational management of filters after sampling. Specifically, the analysis focused on the effect of filter recovery time, i.e., the elapsed time between the end of sampling and filter removal for conditioning and laboratory weighing. Two field scenarios were reproduced: in the first (*daily analysis*), filters were removed within 24 h of sampling completion; in the second (*weekly analysis*), filters were left in the sampler for up to 168 h (7 days) before removal.

A total of 56 datapoints were collected for each experimental stage, resulting in an overall dataset of 336 datapoints for the entire study.

#### Filter conditioning and pre/post sampling handling

Filter conditioning represents one of the most critical steps in gravimetric PM determination, as particulate mass is strongly affected by temperature, relative humidity (RH) and equilibration time.

In this study, all filters were conditioned in a dedicated climatic room at 20 ± 1 °C and 50 ± 5% RH for at least 48 h, in accordance with EN12341:2023. These conditions are consistent with widely adopted international protocols for gravimetric PM analysis and have been shown to minimize mass instability driven by hygroscopic water uptake and volatilization processes (Buonanno et al., 2011; Lacey & Faulkner, 2015).

After sampling, filters remained in the sampler cassette until retrieval to minimize exposure to ambient humidity fluctuations that can affect mass stability. Immediately after removal, each filter was sealed in a clean Petri dish and transported to the conditioning facility. The elapsed time between sampler removal and reconditioning was kept below 1 h, in accordance with good practice recommendations and interlaboratory protocols (EMEP/EEA, 2019). Rapid transfer minimizes volatilization losses, particularly of ammonium nitrate, a known artifact in PM2.5 sampling (Schaap et al., 2004; Viana et al., 2006).

Post-sampling conditioning was again performed for ≥ 48 h under identical T/RH conditions to ensure mass equilibration before weighing. Symmetric pre/post conditioning is essential because inconsistent thermo-hygrometric conditions can cause significant biases in gravimetric PM, especially for fine particles with high semi-volatile content (Watson & Chow, 2011). In line with EN12341:2023, no desiccants were used to avoid excessive drying or chemical interactions that could modify filter mass.

Finally, filters were visually inspected for damage, visible artifacts, fiber separation or deposition irregularities, as recommended in EN12341:2023 and interlaboratory QA/QC protocols (Ballesta et al., 2014). Any filters not meeting integrity or mass stability criteria were excluded.

#### Weighing procedure

The gravimetric determination of filter mass was carried out in accordance with EN12341:2023, which prescribes strict requirements on balance performance, weighing environment, and filter handling in order to ensure measurement repeatability and minimize uncertainty. All weighing operations were performed in a dedicated microbalance laboratory isolated from air turbulence and vibration, following the procedures recommended in international guidelines for high-precision mass measurements (Buonanno et al., 2011).

A microbalance with a readability of 1 µg and automatic internal calibration was used for all gravimetric determinations. Before each weighing session, the balance was leveled and its internal calibration function activated. External calibration with certified E2-class masses was performed at regular intervals to ensure traceability to national standards, consistent with best practices in metrological laboratories (Lacey & Faulkner, 2015).

Filters were handled exclusively with antistatic tweezers and placed gently on the weighing pan to avoid fiber disruption or particle loss. Each filter was weighed at least twice, and the measurement was accepted only when the difference between consecutive readings was ≤ 5 µg, as prescribed by EN12341:2023. If this criterion was not met, the filter was returned to the conditioning room for additional equilibration and subsequently reweighed. This iterative process reduces the risk of recording unstable masses related to insufficient equilibration or environmental transients.

During each weighing session, laboratory blanks were analyzed to detect potential drifts in balance response or environmental conditions. Consistency between blank masses over time (typically < 2–3 µg deviation) was used as an internal quality-check measure, in line with the QA/QC procedures used by major PM monitoring networks (EMEP/EEA, 2019).

#### Storage protocol and definition of the recovery time variable

Proper storage of filters after sampling is essential to minimize mass instability and prevent artifacts that could affect gravimetric PM determination. As required by EN12341:2023, filter handling was performed under controlled conditions to limit thermal and hygroscopic shocks that may influence the final measured mass.

A key operational parameter investigated in this study is the recovery time, defined as the interval between the end of the 24-h sampling period and the removal of the filter from the sampler cassette for conditioning and weighing. EN12341:2023 does not prescribe a specific maximum recovery time.

To evaluate the influence of this variable, two operational scenarios were reproduced:

- daily recovery, with filters removed within 24 h from the end of sampling;
- weekly recovery, with filters left in the sampler cassette for 168 h (7 days).

These conditions reflect common practices in air-quality monitoring networks across European countries, where filter retrieval frequency can vary due to logistical constraints and the widespread adoption of sequential samplers.

After removal from the sampler, filters were visually inspected and transferred to the conditioning facility following the protocol described in Sect. 2.2.1.

#### Flow-rate verification and quality assurance/quality control (QA/QC)

Accurate control of the sampler flow rate is essential to ensure the validity of gravimetric PM measurements, as flow deviations directly affect the sampled air volume and the representativeness of the collected particulate mass. In accordance with EN12341:2023, both low-volume samplers were operated at a nominal flow rate of 2.3 ± 0.1 m^3^ h⁻^1^, and flow performance was regularly verified throughout the monitoring campaigns.

Flow-rate checks were conducted before the start of the campaign, every 14 days during operation, and at the end of the sampling period using a certified electronic flow calibrator traceable to national standards. This verification schedule is consistent with good practice recommendations adopted in regulatory monitoring networks and intercomparison studies (EMEP/EEA, 2019). Samples were excluded from analysis when the average flow rate deviated by more than ± 5% from the nominal value or when the 24-h sampling duration was incomplete, in accordance with EN12341:2023.

Mass stability was confirmed through repeated weighings, as described in Sect. 2.2.2.

### Statistical analysis

Statistical analysis was performed to evaluate the variability of PM10 and PM2.5 concentrations under the different experimental configurations and to quantify the uncertainty associated with each operational variable. Descriptive statistics, including mean, median, standard deviation, interquartile range, and coefficient of variation, were calculated for each dataset to characterize central tendency and dispersion. Data distributions were visualized using histograms and boxplots to assess variability across filter types and recovery time scenarios. Normality was assessed using the Shapiro–Wilk test (Ghasemi & Zahediasl, 2012; Shapiro & Wilk, 1965), while homogeneity of variance was evaluated using Levene’s test (Levene, 1960; Zhou et al., 2023). Depending on distributional properties, group comparisons were performed through parametric methods such as the Student’s *t*-test and one-way ANOVA or through non-parametric alternatives such as the Mann–Whitney *U* test and Kruskal–Wallis test. Post-hoc analyses, including Tukey HSD or Dunn’s test, were applied where appropriate to identify significant differences between experimental conditions. All statistical tests were implemented using standard, validated procedures available in the software packages described below. In addition, correlations between meteorological parameters and PM concentrations were evaluated using Pearson or Spearman correlation coefficients, selected according to the underlying distribution, to examine possible confounding effects or interactions with the investigated operational variables.

Uncertainty estimation was carried out in accordance with the *Guide to the Expression of Uncertainty in Measurement* (GUM). For each variable, the standard uncertainty was calculated from the standard deviation (*s*) of replicate measurements normalized by the square root of the number of observations (*n*), as shown in Eq. (1):

1 $${\mu}_{i}= \frac{s}{\sqrt{n}}$$

where ${\mu}_{i}$ is the standard uncertainty associated with the *i*-th variable.

The combined standard uncertainty was then obtained by propagating the individual contributions under the assumption of independence, following the root-sum-square approach in Eq. (2):

2 $${\mu}_{c}=\sqrt{\sum {{\mu}_{i}}^{2}}$$

where ${\mu}_{c}$​ represents the combined standard uncertainty.

Expanded uncertainty was finally calculated by multiplying the combined standard uncertainty by a coverage factor (k = 2), corresponding to a 95% confidence level, according to the GUM concept reported in Clause 9.2 of EN12341:2023.

In this framework, the uncertainty analysis focuses exclusively on the operational variables intentionally modified in the study, filter material and recovery time, while variability associated with conditioning, storage, weighing procedures and instrument performance was minimized through strict QA/QC controls in accordance with EN12341:2023 and therefore not included in the comparative uncertainty evaluation.

All statistical analyses and data processing were carried out using *Microsoft Excel 365* (Microsoft Corporation, USA) for descriptive statistics and graphical representation of distributions, while *IBM SPSS Statistics v.29* (IBM Corp., USA) and *R software v.4.3.2* (R Foundation for Statistical Computing, Austria) were employed for inferential testing, correlation analysis, and post-hoc comparisons. Uncertainty evaluation, error propagation, and advanced statistical calculations were additionally performed in *Python 3.11* (Python Software Foundation, USA) using libraries such as *NumPy*, *SciPy*, and *pandas*.

## Results and discussion

This section presents and discusses the results obtained from the experimental activity. After outlining the gravimetric measurement chain to frame the methodological context, the effects of the two operational variables investigated, filter material and filter recovery time, are analyzed for both PM10 and PM2.5. It should be noted that PM10 and PM2.5 were sampled during different periods; therefore, their concentration levels are not directly comparable, and each fraction is evaluated independently.

### Gravimetric measurement chain and methodological framework

**Fig. 2:**
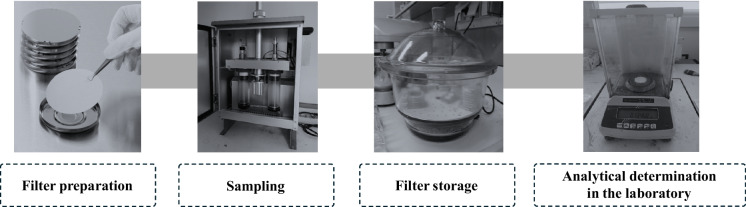
Schematic representation of the gravimetric measurement chain highlighting potential interference units

The gravimetric determination of PM10 and PM2.5 concentrations follows a structured sequence of filter conditioning, sampling, post-sampling storage, and laboratory weighing. Each stage introduces potential interferences that may compromise the reliability of the measurement chain. Figure 2 schematically illustrates this process, highlighting the points most exposed to operational or environmental variability, such as filter conditioning and recovery time. This framework, consistent with EN12341:2023, provided the methodological basis for the present investigation.

As widely recognized in the literature (Buonanno et al., 2011; Lacey & Faulkner, 2015), each phase of the measurement chain may introduce mass artifacts or uncertainty contributions. In the present work, all standardized steps (conditioning, weighing, sampler operation) were kept constant across configurations, allowing the effects of the two non-standard operational variables, filter material and recovery time, to be isolated and quantified.

### Effect of filter material

**Fig. 3:**
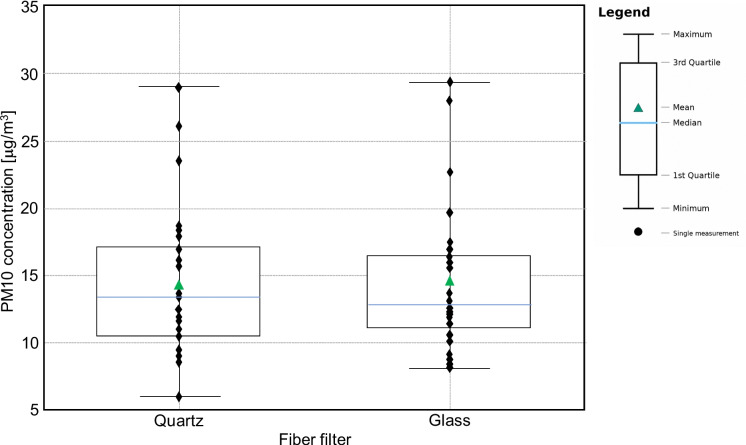
Boxplots of PM10 concentrations values by filter type (glass vs. quartz), showing median, interquartile ranges, mean and single measurements for each filter category

For PM10, mean concentrations derived from glass (14.56 µg m⁻^3^) and quartz (14.29 µg m⁻^3^) filters were nearly indistinguishable (Fig. 3).

The boxplots show overlapping interquartile ranges (IQRs: 11.08–16.41 µg m⁻^3^ for glass and 10.51–17.12 µg m⁻^3^ for quartz) with similar coefficients of variation (CV = 37.58% for glass and 37.63% for quartz).

Statistical tests confirmed no significant differences between the two filter materials (t-test: p = 0.62; Mann–Whitney: p = 0.58). These results indicate that, for coarse particles (PM10), the filter substrate exerts negligible influence on mass concentration. This outcome is consistent with previous intercomparison campaigns (Gualtieri et al., 2009; Putaud et al., 2010), which reported discrepancies below 5% between glass and quartz for PM10.

Consequently, the filter material contributes only marginally to PM10 variability under typical field conditions.

**Fig. 4:**
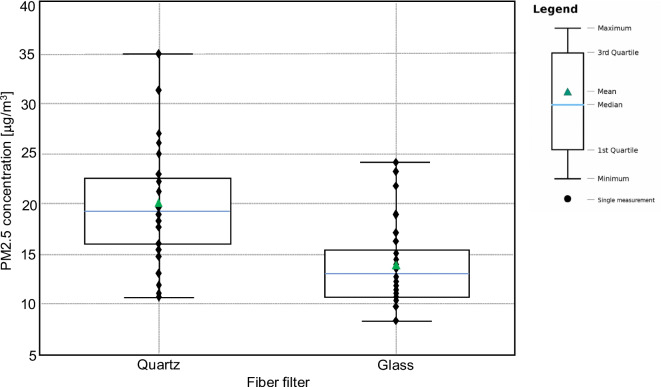
Boxplots of PM2.5 concentrations values by filter type (glass vs. quartz), showing median, interquartile ranges, mean and single measurements for each filter category

In contrast, the PM2.5 results show a strong influence from the filter material. Quartz yielded systematically higher values compared to glass (Fig. 4).

Systematically higher PM2.5 concentrations obtained with quartz filters compared to glass filters were detected. For quartz, the mean concentration was 20.13 µg m^−3^ (median = 19.31 µg m^−3^, range = 10.61–34.90 µg m^−3^, CV = 28.58%), while for glass the mean was lower, 14.04 µg m^−3^ (median = 13.38 µg m^−3^, range = 8.43–24.12 µg m^−3^, CV = 28.94%). The upward shift in values with quartz is evident both in central tendency and in the upper extremes. Statistical testing confirmed the significance of these differences (ANOVA: p < 0.001; Kruskal–Wallis: p < 0.010).

These results align with previous observations by Chow et al. (2010) and Vecchi et al. (2009), which showed that quartz filters offer superior thermal stability. This mechanism explains the systematically higher masses and greater variability observed in our study. Consequently, PM2.5 determinations appear disproportionately sensitive to the choice of substrate, with quartz filters amplifying variability beyond the levels generally considered acceptable in regulatory monitoring.

The magnitude of the differences observed here (up to ~ 45–50% between configurations) is consistent with documented positive artifacts associated with quartz filters due to OC adsorption, as well as negative artifacts for glass fiber filters due to nitrate and semi-volatile loss (Schaap et al., 2004; Viana et al., 2006). Therefore, the choice of substrate emerges as a dominant source of operational variability for PM2.5, reinforcing the need to explicitly consider filter material in uncertainty assessments and monitoring guidelines.

### Effect of recovery time

**Fig. 5:**
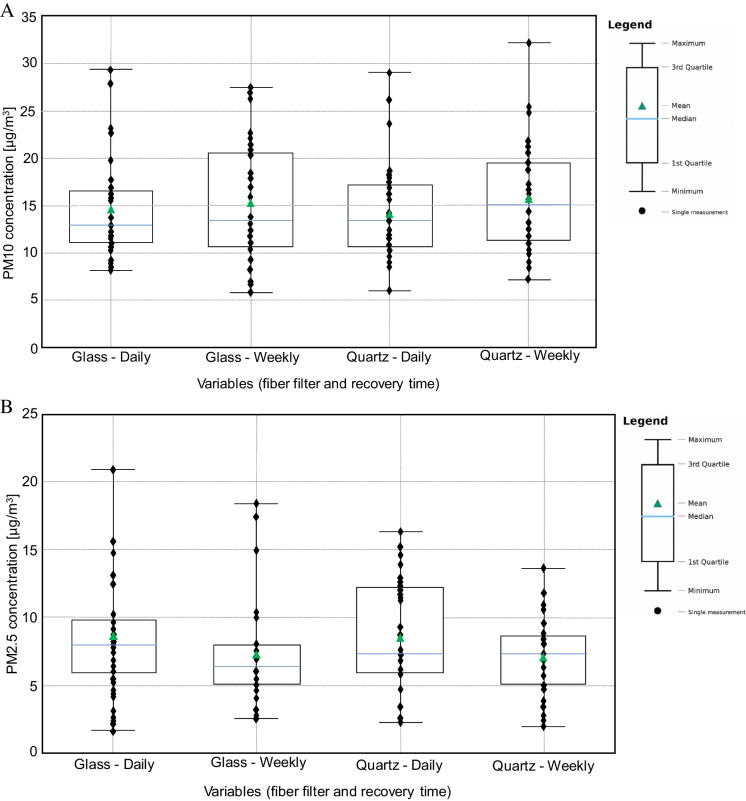
(**A**) Boxplots of PM10 concentrations values under daily vs weekly recovery time, for both filter types (glass vs. quartz), showing median, interquartile ranges, mean and single measurements for each filter category. (**B**) Boxplots of PM2.5 concentrations under daily vs weekly recovery time, for both filter types (glass vs. quartz), showing median, interquartile ranges mean and single measurements for each filter category

Different protocols on recovery time revealed contrasting effects between PM10 and PM2.5. For PM10, weekly recovery yielded systematically higher concentrations than daily recovery. In quartz filters, the mean increased from 14.29 µg m⁻^3^ (daily) to 15.76 µg m⁻^3^ (weekly), representing a relative difference of + 10.25% (Fig. 5A). For glass, the increase was smaller (+ 4.54%, from 14.56 µg m⁻^3^ to 15.22 µg m⁻^3^). Statistical comparisons confirmed significance for quartz (paired t-test: p = 0.0012) but not for glass (p = 0.2000). This behavior is consistent with the hypothesis of additional mass gain during prolonged exposure, possibly due to the deposition of coarse resuspended particles.

Similar tendencies have been reported in previous studies, where extended filter residence times led to small but systematic increases in coarse-particle mass due to hygroscopic uptake or redeposition of larger particles (Allen et al., 2017). These results confirm that, for PM10, the recovery time introduces only a modest bias that typically remains within expected operational variability.

For PM2.5, the opposite trend to PM10 was observed. Daily recovery yielded systematically higher concentrations than weekly recovery for both filter materials (Fig. 5B). On quartz, the mean concentration decreased from 8.78 µg m⁻^3^ (daily) to 7.31 µg m⁻^3^ (weekly), corresponding to a relative reduction of 16.68%. A similar pattern was found for glass, where concentrations dropped from 8.72 µg m⁻^3^ (daily) to 7.45 µg m⁻^3^ (weekly), a reduction of 14.54%. Coefficients of variation were higher for glass (55.21% daily; 52.06% weekly) compared to quartz (45.54% daily; 42.20% weekly), indicating greater variability. Statistical analysis confirmed these differences as highly significant (p < 0.0043 for quartz; p = 0.0074 for glass, both parametric and non-parametric tests). This pattern strongly suggests volatilization of semi-volatile species (e.g., ammonium nitrate, organics) during extended residence in the sampler, as documented by Schaap et al. (2004) and Viana et al.(2006). The reductions observed here (14–17%) fall within the ranges reported for volatilization of ammonium nitrate and semi-volatile organic compounds during storage or delayed handling. Numerous studies have quantified analogous losses of 10–30% depending on ambient temperature and humidity (Schaap et al., 2004; Chiu & Carlton, 2023). This behavior underscores the high instability of fine particulate mass under non-ideal post-sampling conditions and highlights the importance of rapid filter retrieval for PM2.5 monitoring.

Thus, while PM10 appears sensitive primarily to accumulation processes under weekly recovery, PM2.5 is strongly affected by volatilization losses, emphasizing the importance of rapid filter retrieval for fine-particle gravimetry.

### Comparative influence of filter material and recovery time

The joint evaluation of filter material and recovery time highlights a fundamental asymmetry between coarse and fine particle fractions.

**Fig. 6:**
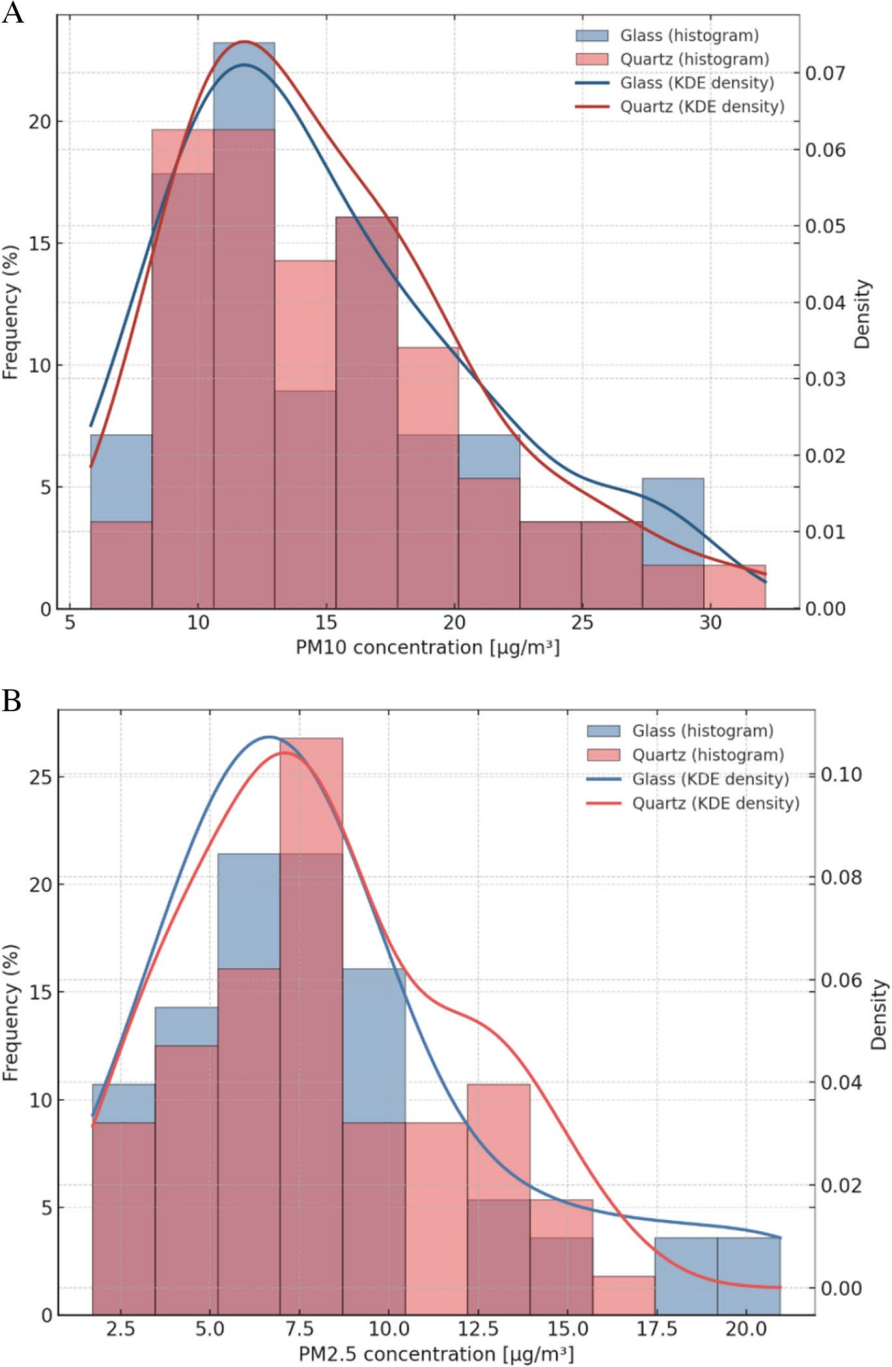
(**A**) PM10 histograms by filter material. (**B**) PM2.5 histograms by filter material

For PM10, the choice of filter material exerted negligible influence. Mean concentrations were 14.89 µg m⁻^3^ for glass and 15.03 µg m⁻^3^ for quartz (Fig. 6A). The histograms confirm a nearly identical shape, with both filters showing modal classes in the 10.40–14.70 µg m⁻^3^ range and maximum values reaching 29.30 µg m⁻^3^ (glass) and 32.10 µg m⁻^3^ (quartz). Frequency is reported on the left y-axis (%), while kernel density estimates (KDE) are shown on the right y-axis. Kernel density estimates (KDE) represent a non-parametric method used to approximate the probability density function of a dataset, providing a smoothed continuous representation of the underlying data distribution. Overlapping KDE curves indicate similar distributions, while shifts or changes in shape reflect differences in concentration levels and variability. The coefficients of variation were moderate but comparable (39.36% for glass, 37.46% for quartz), and no statistically significant differences were detected (t-test: p = 0.5700; Mann–Whitney: p = 0.8400).

When recovery time was considered, weekly sampling yielded slightly higher concentrations than daily sampling. On quartz filters, the mean increased from 13.60 µg m⁻^3^ (daily) to 15.40 (weekly) µg m⁻^3^ (weekly), corresponding to a relative difference of + 13%. On glass, the increase was more modest (+ 3%, from 15.00 to 15.50 µg m⁻^3^). Weekly recovery thus appears to reduce potential losses or allow minor additional deposition during prolonged residence in the sampler. Nevertheless, overall variability remained low, with ANOVA confirming no significant main effects (p > 0.0500).

In contrast, PM2.5 was strongly affected by both filter material and recovery protocol. Quartz filters systematically produced higher concentrations and broader variability compared to glass (Figs. 4 and 6B). The mean concentration on quartz was 15.30 µg m⁻^3^, against 10.50 µg m⁻^3^ on glass, corresponding to a relative difference of about + 46%. The histograms confirm this divergence, with quartz showing a wider spread and frequent values above 20 µg m⁻^3^, while glass distributions were more clustered in the 8–14 µg m⁻^3^ range.

Recovery time further modulated these outcomes: daily recovery yielded higher values than weekly. The most divergent configurations were quartz-daily (mean 15.30 µg m⁻^3^) and glass-weekly (mean 10.50 µg m⁻^3^), whose difference exceeded 45%.

Statistical analyses confirmed these interaction patterns. Two-way ANOVA revealed highly significant main effects of both filter material (p < 0.0010) and recovery time (p < 0.0100) for PM2.5, whereas interaction terms were not significant for PM10. Non-parametric post-hoc tests (Dunn’s test) corroborated that quartz-daily datasets consistently ranked higher than quartz-weekly and any glass-based condition, with mean rank differences > 20%.

These outcomes are consistent with intercomparison studies in European monitoring networks, where differences in operational practices contributed to uncertainties exceeding the 5% target defined in EN12341:2023 (European Commission. Joint Research Centre., 2025). The evidence presented here confirms that fine particles are much more vulnerable to operational artifacts than coarse fractions, an aspect that must be explicitly recognized and accounted for in regulatory frameworks.

The observed asymmetry between PM10 and PM2.5 is fully coherent with the composition-dependent nature of sampling artifacts. Coarse PM contains primarily inert or mineral particles, whereas fine PM includes volatile and semi-volatile fractions susceptible to adsorption, chemical reaction, or evaporation. This explains why operational differences that are negligible for PM10 become dominant uncertainty sources for PM2.5. Similar findings have emerged in European monitoring network audits (European Commission. Joint Research Centre., 2025), where operational variability contributed disproportionately to uncertainties in PM2.5.

### Statistical analysis of variability and group differences

Table 3 summarizes the statistical analyses performed for PM10 and PM2.5 under the different investigated combinations over the entire monitoring period.

**Table 3:** Summary of the statistical analysis for PM10 and PM2.5 under different investigated combinations

PM fraction	Combinations	Mean [µg m^−3^]	IQR* [µg m^−3^]	Statistical test	
				type	results
PM10	• Daily recovery time • Quartz fiber filter	14.29	11.94–16.07	Shapiro–Wilk	p > 0.10
	• Weekly recovery time • Quartz fiber filter	15.76	14.02–18.24	Parametric	p > 0.05
	• Daily recovery time • Glass fiber filter	14.56	12.60–17.48	Tukey HSD post-hoc	no significant differences
	• Weekly recovery time • Glass fiber filter	15.22	9.04–16.68		
PM2.5	• Daily recovery time • Quartz fiber filter	8.78	5.96–12.33	Shapiro–Wilk	p < 0.05
	• Weekly recovery time • Quartz fiber filter	7.31	5.91–9.93	Mann–Whitney and Kruskal–Wallis	p < 0.01
	• Daily recovery time • Glass fiber filter	8.72	5.24–8.74	Tukey HSD post-hoc	Quartz–daily significantly higher
	• Weekly recovery time • Glass fiber filter	7.45	5.16–8.09		

*IQR = Interquartile Range

**CV = Coefficient of Variation

The results confirm that PM2.5 exhibits markedly broader dispersion than PM10, particularly under quartz-daily conditions. Glass filters showed systematically lower and more compact values.

By comparison, PM10 distributions were more stable and less dispersed.

Overall, these results further confirm that variability is primarily driven by PM2.5 and filter material, with quartz enhancing both absolute concentrations and dispersion. PM10 instead shows greater robustness to filter substrate and recovery protocol, maintaining narrower and more homogeneous distributions with CV consistently below 40%.

**Fig. 7:**
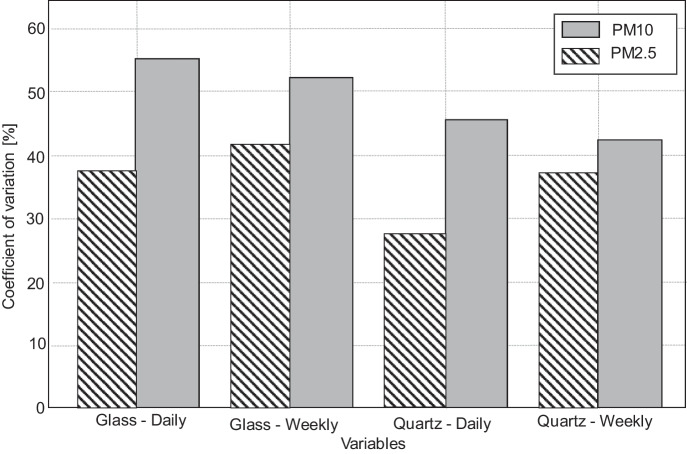
Coefficients of variation (CV%) for PM10 and PM2.5 across filter and recovery time

Descriptive statistics confirmed these patterns. Coefficients of variation (CV%) for PM10 and PM2.5 were represented in Fig. 7. This greater variability in fine particles is consistent with their enhanced sensitivity to artifacts related to filter-substrate interactions and environmental conditions. Shapiro–Wilk tests confirmed that PM2.5 datasets frequently deviated from Gaussian distributions (p < 0.05), whereas PM10 datasets generally satisfied normality assumptions (p > 0.10).

For PM2.5, both Mann–Whitney and Kruskal–Wallis tests yielded highly significant differences between filter types (p < 0.01) and recovery times (p < 0.01). In contrast, PM10 datasets did not exhibit statistically significant differences (p > 0.05) for either factor. Post-hoc analyses (Tukey HSD and Dunn’s test) identified the quartz-daily configuration as systematically higher than quartz-weekly and all glass-based conditions, with mean rank differences exceeding 20%. These outcomes confirm that fine fractions are disproportionately affected by operational variables, whereas coarse fractions remain relatively stable.

Overall, the combined evidence from descriptive, normality, variance-homogeneity, and hypothesis-testing analyses demonstrates that variability in gravimetric PM2.5 determinations is not random noise but a systematic effect of filter material and recovery time, in agreement with prior interlaboratory studies (Ballesta et al., 2014).

The significant group differences observed for PM2.5, combined with non-Gaussian distributions and high CV values, reinforce the interpretation that the observed variability is not random noise but a systematic result of operational settings. These results are consistent with intercomparison campaigns showing that PM2.5 is intrinsically more prone to artifacts during sampling and handling (Ballesta et al., 2014), especially when semi-volatile species are present.

### Uncertainty budget and methodological implications

The uncertainty budget is summarized in Table 4.

**Table 4:** Uncertainty budget for PM10 and PM2.5 across filter materials and recovery times

PM fraction	Stage	Non-standard variables	C_mean_ [μg m^−3^]	Standard Uncertainty	
				μ_i, mean_ [μg m^−3^]	% of mean
PM10	I	Filter material	14.43	0.70	4.85%
	II	Recovery time [testing glass FIBER filter]	14.89	1.19	7.79%
	III	Recovery time [testing quartz FIBER filter]	15.03	1.11	7.38%
PM2.5	I	Filter material	17.08	3.09	18.09%
	II	Recovery time [testing glass FIBER filter]	8.10	1.07	13.20%
	III	Recovery time [testing quartz FIBER filter]	8.21	1.08	13.15%

For PM10, uncertainty contributions were modest: filter material accounted for 4.85% (0.70 µg m⁻^3^) of mean concentrations, while recovery time added 7.79% (1.19 µg m⁻^3^) when testing glass fiber filters and 7.38% (1.11 µg m⁻^3^) when testing quartz fiber filters. These values remain within the 10% data quality objective defined in EU reference guidelines.

For PM2.5, however, uncertainty was substantially higher: filter material contributed 18.09% (3.09 µg m⁻^3^), and recovery time contributed 13.20% (1.07 µg m⁻^3^) on glass filters and 13.15% (1.08 µg m⁻^3^) on quartz filters, yielding total propagated uncertainties approaching 20%. The disproportionate influence of filter material on PM2.5 is particularly concerning. Under the revised EU Directive 2024/2881, the annual-mean limit for PM2.5 will be reduced to 10 µg m⁻^3^ by 2030. An uncertainty margin of 18% could therefore shift the apparent concentration large enough to change compliance status. This demonstrates that operational variables, though not explicitly accounted for in EN12341:2023, may undermine regulatory reliability if left unaddressed. These results corroborate findings from other field trials (Allen et al., 2017; Viana et al., 2006), where operational choices such as filter handling and storage dominated overall uncertainty. These findings place the operational uncertainty of PM2.5 within the upper range of values documented in previous GUM-based and field studies (typically 12–20%). Given the upcoming tightening of limit values under Directive (EU) 2024/2881, such levels of uncertainty may meaningfully influence compliance decisions near critical thresholds, highlighting the need for more standardized practices for filter handling and recovery times.

## Critical synthesis, perspectives and conclusion

The research systematically investigated the influence of filter material and recovery time on gravimetric determinations of PM10 and PM2.5, focusing on their contribution to measurement variability and overall uncertainty. The findings highlight a structural asymmetry between the two fractions: PM10 measurements were relatively stable, with recovery time exerting a modest influence and filter type largely negligible, whereas PM2.5 determinations were markedly sensitive to both variables. Quartz filters consistently yielded higher and more variable concentrations than glass, and weekly recovery systematically underestimated mass relative to daily recovery, with differences quantified at 16.70% for quartz (from 8.78 to 7.31 µg m⁻^3^) and 14.50% for glass (from 8.72 to 7.45 µg m⁻^3^).

The reconstructed uncertainty budget confirmed these patterns, with PM2.5 uncertainties reaching 18.09% for filter material and 13.20–13.15% for recovery time, well above typical data-quality objectives, while PM10 uncertainties remained between 4.85% and 7.79%. Such values suggest that operational choices, often considered minor, can introduce variability comparable to or greater than instrumental errors. Furthermore, these levels of variability are substantial in the context of regulatory thresholds: under the forthcoming Directive (EU) 2024/2881, which lowers annual PM2.5 limits to 10 µg m⁻^3^, an uncertainty margin of 18.09% corresponds to a deviation of 1.81 µg m⁻^3^, large enough to critically affect compliance assessment.

The integrated evaluation of filter material, recovery time, statistical variability, and uncertainty propagation provides a comprehensive picture of how operational factors influence gravimetric PM determinations. The evidence consistently shows that PM10 measurements are comparatively robust, whereas PM2.5 determinations are highly sensitive to filter substrate and handling protocols, leading to propagated uncertainties that may exceed regulatory tolerance.

These findings highlight that operational variability can exert an influence comparable to, or even greater than, instrumental uncertainties. Unlike previous studies that primarily focused on individual artifacts or specific measurement configurations, the present work provides a systematic quantification of the contribution of operational variables within an integrated uncertainty framework under field conditions relevant to European monitoring networks. Accordingly, harmonization of filter media across monitoring networks, minimization of recovery times, and the systematic incorporation of humidity corrections should be prioritized. In addition, explicit reporting of uncertainty budgets should be encouraged in regulatory assessments, particularly for borderline cases near tightened EU thresholds.

From a regulatory perspective, the present outcomes, though obtained within a specific concentration range (2.09–34.90 µg m⁻^3^ for PM2.5; 6.02–32.14 µg m⁻^3^ for PM10), suggest that further targeted studies are needed to assess whether filter type and recovery time should be more explicitly integrated into standardization and quality assurance procedures.

These considerations should also be interpreted in the context of international regulatory differences, as U.S. reference methods mandate the use of PTFE filters to minimize sampling artifacts, whereas the European framework allows multiple filter media under a performance-based approach (EN12341:^2023^; EMEP/EEA, 2019). In this context, the results of the present study further support the need for explicit consideration of filter-related uncertainty in EU monitoring practices.

It is important to acknowledge certain limitations. First, only quartz and glass fiber filters were tested; PTFE filters, recommended in several regulatory frameworks and less prone to artifacts, were not included. Second, the absence of chemical composition data limits mechanistic interpretation of observed differences. Third, PM10 and PM2.5 were sampled in different periods, preventing meaningful comparison of absolute concentration levels across fractions. Finally, the study was conducted at a single site, and results may vary across climates or source environments.

Overall, these findings contribute to a more comprehensive understanding of the gravimetric measurement chain, highlighting where refinements and further investigations are most needed. By linking methodological aspects of particulate-matter measurement to uncertainty quantification, this research supports the improvement of data quality, comparability and robustness in regulatory and research-oriented monitoring networks, particularly in the context of increasingly stringent regulatory requirements.

## Data Availability

The raw data used for analysis will be provided by the corresponding author upon request.

## Abbreviations

PM: Particulate matter
PM10: Particulate matter with aerodynamic diameter ≤ 10 µm
PM2.5: Particulate matter with aerodynamic diameter ≤ 2.5 µm
NOx: Nitrogen oxides
SO₂: Sulfur dioxide
CO: Carbon monoxide
O₃: Ozone
EU: European Union
WHO: World Health Organization
EN12341:2023: European Standard for gravimetric PM10 and PM2.5 determination
GAW: Global Atmosphere Watch
GUM: Guide to the Expression of Uncertainty in Measurement
CV: Coefficient of variation
SD: Standard deviation
IQR: Interquartile range
KDE: Kernel density estimate
RH: Relative humidity
T: Temperature
PTFE: Polytetrafluoroethylene
QA/QC: Quality assurance/quality control
ANOVA: Analysis of variance
SPSS: Statistical Package for the Social Sciences
EPA: Environmental Protection Agency
FRM: Federal Reference Method
FEM: Federal Equivalent Method
TSP: Total suspended particulate
JRC: Joint Research Centre (European Commission)
EMEP: Cooperative Programme for Monitoring and Evaluation of the Long-range Transmission of Air Pollutants in Europe
Uᵢ: Standard uncertainty associated with the i-th variable
Uᶜ: Combined standard uncertainty
k: Coverage factor
